# Supplementary material for: Time trend, social vulnerability, and identification of risk areas for tuberculosis in Brazil: An ecological study
Source: PLoS One. 2022 Jan 25;17(1):e0247894. doi: 10.1371/journal.pone.0247894 (PMC8789117; doi:10.1371/journal.pone.0247894)
Supplement: S1 Table — (DOCX) [file pone.0247894.s001.docx]

**S1 Table.** Performance of these two models - OLS and SLM.

| **Criteria** | **Block 1- Social Vulnerability Index** | | **Block 2- Subindices of the Social Vulnerability Index** | | **Block 3- Variables of SVI Urban Infrastructure** | | **Block 4- Variables of SVI Human Capital** | | **Block 5- Variables of SVI Income and Work** | | **Block 6- Other indicators of social vulnerability** | |
| --- | --- | --- | --- | --- | --- | --- | --- | --- | --- | --- | --- | --- |
|  | **OLS** | **SLM** | **OLS** | **SLM** | **OLS** | **SLM** | **OLS** | **SLM** | **OLS** | **SLM** | **OLS** | **SLM** |
| R2 | 0.023 | 0.355 | 0.022 | 0.3557 | 0.048 | 0.3625 | 0.082 | 0.362 | 0.116 | 0.371 | 0.127 | 0.374 |
| Log likelihood | -24871.7 | -23910.4 | -24895.9 | -23908.9 | -24821.3 | -23872.8 | -2479.3 | -23860.2 | -24614.5 | -23816.4 | -24577.5 | -23796 |
| Akaike (AIC) | 49787.7 | 47826.9 | 49799.9 | 47827.8 | 49650.5 | 47755.5 | 49456.5 | 47740.3 | 49241.0 | 47646.8 | 49185.0 | 47624 |
| Schwarz (SBC) | 49800.9 | 47826.7 | 49826.4 | 47861.0 | 49677.0 | 47788.6 | 49516.1 | 47806.6 | 49280.0 | 47693.2 | 49284.3 | 47729.2 |
| I Moran | 0.3514 (P<0.001) | - | 0.3470  (P<0.001) | - | 0.3308  (P<0.001) | - | 0.3063  (P<0.001) | - | 0.2891  (P<0.001) | - | 0.2707  (P<0.001) | - |

**Legend**: OLS- Ordinary least squares; SLM- Spatial lag model
