# Supplementary material for: Time trend, social vulnerability, and identification of risk areas for tuberculosis in Brazil: An ecological study
Source: PLoS One. 2022 Jan 25;17(1):e0247894. doi: 10.1371/journal.pone.0247894 (PMC8789117; doi:10.1371/journal.pone.0247894)
Supplement: S2 Table — Brazil, 2001–2017. (DOCX) [file pone.0247894.s002.docx]

**S2 Table.** Incidence rate of tuberculosis per 100,000 inhabitants by Social Vulnerability Index classification and subindices. Brazil, 2001-2017.

| **Level of social vulnerability** | **2001** | **2002** | **2003** | **2004** | **2005** | **2006** | **2007** | **2008** | **2009** | **2010** | **2011** | **2012** | **2013** | **2014** | **2015** | **2016** | **2017** |
| --- | --- | --- | --- | --- | --- | --- | --- | --- | --- | --- | --- | --- | --- | --- | --- | --- | --- |
| Overall SVI |  |  |  |  |  |  |  |  |  |  |  |  |  |  |  |  |  |
| Very low | 16.61 | 21.29 | 21.67 | 20.60 | 18.92 | 19.00 | 20.48 | 20.96 | 19.27 | 19.43 | 20.49 | 19.42 | 18.70 | 18.22 | 17.76 | 18.94 | 22.10 |
| Low | 20.11 | 26.83 | 26.72 | 25.09 | 25.11 | 24.99 | 24.31 | 23.97 | 23.58 | 23.87 | 23.59 | 23.03 | 23.71 | 23.41 | 22.00 | 23.35 | 24.86 |
| Medium | 24.91 | 30.37 | 31.48 | 31.91 | 30.38 | 31.84 | 29.65 | 28.15 | 26.85 | 26.56 | 27.29 | 25.70 | 25.71 | 23.60 | 22.72 | 24.01 | 25.94 |
| High | 27.89 | 33.95 | 35.22 | 35.09 | 35.79 | 34.34 | 31.05 | 29.60 | 29.89 | 28.37 | 29.40 | 27.75 | 24.92 | 23.58 | 23.55 | 23.80 | 23.76 |
| Very high | 30.01 | 33.65 | 35.99 | 34.33 | 36.75 | 34.68 | 33.64 | 30.83 | 31.84 | 26.95 | 28.39 | 25.40 | 24.69 | 22.90 | 22.00 | 22.24 | 24.17 |
| SVI Urban Infrastructure |  |  |  |  |  |  |  |  |  |  |  |  |  |  |  |  |  |
| Very low | 20.01 | 25.60 | 26.55 | 25.86 | 25.24 | 24.67 | 23.80 | 23.33 | 22.31 | 21.78 | 22.49 | 21.03 | 20.77 | 20.12 | 19.16 | 20.19 | 21.34 |
| Low | 26.02 | 32.46 | 32.15 | 31.72 | 31.71 | 31.51 | 29.37 | 28.12 | 28.65 | 27.34 | 28.02 | 27.11 | 26.04 | 24.20 | 23.39 | 24.09 | 25.79 |
| Medium | 26.46 | 32.23 | 33.08 | 31.70 | 32.66 | 33.81 | 31.63 | 29.82 | 29.28 | 28.45 | 28.48 | 27.78 | 26.67 | 25.28 | 22.76 | 25.25 | 25.48 |
| High | 32.46 | 36.27 | 37.67 | 36.56 | 37.56 | 36.18 | 36.02 | 34.34 | 34.52 | 34.16 | 34.79 | 31.88 | 31.81 | 29.76 | 31.66 | 31.10 | 35.29 |
| Very high | 29.50 | 35.24 | 38.25 | 37.26 | 38.15 | 38.15 | 35.03 | 31.87 | 32.93 | 28.26 | 29.32 | 27.10 | 25.98 | 24.57 | 24.55 | 24.19 | 26.91 |
| SVI Human Capital |  |  |  |  |  |  |  |  |  |  |  |  |  |  |  |  |  |
| Very low | 11.97 | 16.20 | 16.06 | 16.69 | 14.77 | 15.75 | 15.90 | 16.32 | 18.05 | 17.76 | 20.72 | 20.38 | 15.98 | 15.96 | 15.44 | 15.56 | 21.62 |
| Low | 18.63 | 24.51 | 24.67 | 22.90 | 22.06 | 23.34 | 24.26 | 23.73 | 23.12 | 23.27 | 23.77 | 22.75 | 22.80 | 22.46 | 21.60 | 24.00 | 26.48 |
| Medium | 21.96 | 28.27 | 28.31 | 27.72 | 27.04 | 27.55 | 26.18 | 25.88 | 24.97 | 25.67 | 25.12 | 24.56 | 24.99 | 24.14 | 23.84 | 24.04 | 25.59 |
| High | 23.96 | 31.12 | 31.36 | 31.61 | 32.60 | 31.05 | 28.32 | 27.03 | 26.41 | 24.73 | 26.16 | 23.64 | 23.73 | 21.73 | 20.35 | 21.82 | 22.73 |
| Very high | 29.80 | 33.75 | 36.27 | 35.17 | 35.69 | 34.38 | 32.24 | 30.10 | 30.27 | 27.51 | 28.51 | 26.71 | 24.69 | 23.19 | 22.32 | 22.58 | 23.52 |
| SVI Income and Work |  |  |  |  |  |  |  |  |  |  |  |  |  |  |  |  |  |
| Very low | 22.41 | 26.76 | 26.64 | 25.67 | 23.63 | 22.89 | 23.84 | 25.05 | 23.09 | 23.38 | 24.75 | 23.66 | 24.16 | 23.08 | 22.01 | 22.44 | 25.27 |
| Low | 21.08 | 27.54 | 27.12 | 25.73 | 25.41 | 26.13 | 26.90 | 26.30 | 25.11 | 25.74 | 25.51 | 25.77 | 24.95 | 24.39 | 23.77 | 25.11 | 27.90 |
| Medium | 20.83 | 26.60 | 27.71 | 27.13 | 26.12 | 26.09 | 24.68 | 24.15 | 24.37 | 24.31 | 24.88 | 22.57 | 23.73 | 23.48 | 21.86 | 23.08 | 25.15 |
| High | 24.38 | 30.83 | 32.32 | 31.41 | 32.90 | 31.63 | 28.05 | 26.84 | 26.22 | 24.78 | 25.77 | 24.24 | 24.53 | 22.00 | 20.47 | 22.38 | 22.46 |
| Very high | 28.33 | 32.96 | 34.48 | 34.11 | 34.54 | 33.89 | 31.66 | 29.35 | 29.55 | 26.63 | 27.61 | 25.48 | 23.08 | 21.55 | 21.65 | 21.56 | 22.26 |

**Legend:** SVI- Social Vulnerability Index.
